# Sarcoidosis in Northern Ontario hard‐rock miners: A case series

**DOI:** 10.1002/ajim.23333

**Published:** 2022-02-14

**Authors:** L. Christine Oliver, Paul Sampara, Donna Pearson, Janice Martell, Andrew M. Zarnke

**Affiliations:** ^1^ Dalla Lana School of Public Health, Division of Occupational and Environmental Health University of Toronto Toronto Ontario Canada; ^2^ The Occupational Health Clinics for Ontario Workers Sudbury Ontario Canada; ^3^ Department of Kinesiology and Health Sciences, School of Kinesiology and Health Sciences Laurentian University Sudbury Ontario Canada; ^4^ Center for Research in Occupational Safety and Health Laurentian University Sudbury Ontario Canada

**Keywords:** clinical phenotype, granulomatous disease, hard‐rock miner, McIntyre Powder, respirable crystalline silica, sarcoidosis

## Abstract

Sarcoidosis is a rare multisystem granulomatous disease traditionally considered to be of unknown etiology. The notion that sarcoidosis has no known cause is called into question with the increasing number of case reports and epidemiologic studies showing associations between occupational exposures and disease published in the past 10–20 years. Occupational exposures for which associations are strongest and most consistent are silica and other inorganic dusts, World Trade Center (WTC) dust, and metals. Occupations identified as at‐risk for sarcoidosis include construction workers; iron‐foundry and diatomaceous earth workers; WTC emergency responders; and metal workers. We report here 12 cases of sarcoidosis in a cohort of hard‐rock miners in Northern Ontario, Canada. To our knowledge sarcoidosis has not been reported previously in hard‐rock miners. The cases are all male and Caucasian, with average age 74 years. At the time of diagnosis, two were never smokers; six, former smokers; and four, current smokers. Five have extrapulmonary sarcoidosis: two cardiac and three endocrine (hypercalciuria). Using occupational histories and air sampling data from the gold, uranium, and base‐metal mines in which they worked, we examined exposure of each case to respirable crystalline silica (RCS). The annual mean RCS exposure for the 12 cases was 0.14 mg/m^3^ (range: 0.06–1.3 mg/m^3^); and the mean cumulative RCS exposure was 1.93 mg/m^3^ years (range: 0.64–4.03 mg/m^3^ years). We also considered their exposure to McIntyre Powder, an aluminum powder used for silicosis prophylaxis.

## 1 INTRODUCTION

Sarcoidosis is a rare multisystem granulomatous disease. First described by Jonathan Hutchinson in 1877 and named “sarkoid of the skin” by Caesar Boeck in 1899 because of its resemblance to sarcoma, sarcoidosis is an immunologically mediated disorder affecting primarily the lungs and lymph nodes.^1^, ^2^, ^3^ Other organs affected include liver, spleen, heart, eyes, and skin. The formation of noncaseating granulomas is the pathologic hallmark of the disease.^4^ Major diagnostic criteria have been set forth by the American Thoracic Society.^5^ These are: (1) consistent clinical presentation, (2) noncaseating granulomas in one or more tissue samples, and (3) exclusion of other diseases. For the most part sarcoidosis is a sporadic (nonfamilial) disease, occurring in families in 3.6%–9.6% of cases.^6^, ^7^

Sarcoidosis has long been considered a disease of unknown cause, that is, idiopathic. Over the past 20 years a number of case reports, epidemiologic studies of exposed workers, and scientific reviews have been published that show associations between certain workplace exposures and the development of sarcoidosis. Cases of sarcoidosis have been reported in a tunnel worker, a denim sandblaster, iron‐foundry workers, and a plasterer, all exposed to silica.^8^, ^9^, ^10^, ^11^ In 1998 Rafnsson et al.^12^ published the results of a case‐control study showing increased risk of sarcoidosis in a cohort of diatomaceous earth workers in Iceland exposed to cristobalite and quartz. Increased risk for disease has been observed in construction and iron foundry workers, and in World Trade Center (WTC) emergency responders.^13^, ^14^, ^15^, ^16^, ^17^ Scientific reviews of occupational exposures and sarcoidosis have been conducted by Newman and Newman,^18^ and more recently by Oliver and Zarnke.^19^ The strongest and most consistent associations have been observed for silica, WTC dust, and metals.^8^, ^9^, ^10^, ^11^, ^12^, ^13^, ^14^, ^15^, ^16^, ^17^, ^20^, ^21^, ^22^

The purpose of this report is to describe a series of 12 cases of sarcoidosis in a group of hard‐rock miners in Northern Ontario, Canada. We believe that the occurrence of 12 cases of this rare disease in a group with occupational exposure to silica and metal dusts is consistent with previously reported associations and noteworthy. To our knowledge, this is the first such report in hard‐rock miners.

There are three types of hard‐rock mines in Ontario: gold, uranium and other base metal (i.e., nickel, copper, zinc). A historical perspective is helpful in assessing dust exposures incurred by hard‐rock miners over time. Before 1980 dust concentrations in Ontario mines were measured with a hand‐held instrument called a konimeter, an instantaneous dust sampler having a sampling duration of approximately one‐third of a second. Collected dust samples were counted under a microscope and expressed as the number of particles per unit volume of air (particles per cubic centimeter [ppcc]). The main purpose for these dust measurements was to assess the effectiveness of ventilation and other dust control methods.

In the mid‐1970s personal exposures to respirable dust and respirable crystalline silica (RCS) began to be measured using portable, size‐selective samplers and reported in gravimetric units of milligrams per cubic meter (mg/m^3^). Verma et al.^23^ derived conversion factors to allow for the conversion of the earlier ppcc values to mg/m^3^. It is against this backdrop that our series of sarcoidosis cases is presented.

## 2 METHODS

### 2.1 Study population

The sarcoidosis cases are members of a group of hard‐rock miners exposed to a finely ground aluminum powder (McIntyre Powder [MP]) used by mining companies for silicosis prophylaxis during the period 1943–1979.^24^, ^25^ Gold and uranium miners were more likely to be exposed than workers in other types of mines. This practice was discontinued in 1979 after it was found to be ineffective in preventing silicosis.

### 2.2 Data collection

#### 2.2.1 Medical and occupational history

The cohort was assembled by the recruitment of miners exposed to MP.^26^ A voluntary registry of MP‐exposed miners was established based on research on the use of MP in Northern Ontario mines. In 2016 intake clinics were held in two mining communities to obtain demographic information, medical and family histories, and occupational and environmental exposure histories. Participation was invited from MP registrants and members of the two mining communities.

The intake clinics were staffed by a multidisciplinary group of health professionals: experienced physicians and occupational health nurses, industrial hygienists, and ergonomists. The clinics were carried out with the cooperation and assistance of representatives of labor and Ontario‐government‐funded organizations and agencies. One of these organizations, Occupational Health Clinics for Ontario Workers (OHCOW), was instrumental in providing financial and organizational support for the intake clinics and for the collection, organization, and subsequent analysis of the information collected.

Intake clinic staff administered detailed questionnaires designed to obtain health information, a chronologic lifetime work history, and job‐specific exposure information. Where the miner was deceased or incapacitated due to illness, interviews were conducted with next‐of‐kin or the executor of the miner's estate (often the same). Mandatory training was held for interviewers.

Registration of MP‐exposed miners has continued following the intake clinics and is ongoing in 2021. Telephone interviews are conducted by an experienced occupational health nurse (D. P.) and questionnaires administered. In addition, medical records and workers' compensation claims information are requested from treating physicians and the Workplace Safety and Insurance Board (WSIB), respectively. Mining employment history and MP exposure status provided by each of the miners was confirmed using the Ontario Mining Master File (MMF) record maintained by the WSIB and a list of mines that were officially documented by the McIntyre Research Foundation as using MP. The list of mines is appended to the MMF (Appendix F of the Coding Manual—MMF).^25^ An MP Project database (MPPD) has been established using data collected at the intake clinics and subsequently.

### 2.3 Case definition

Cases in the series are miners in the MPPD who answered “yes” to the question “Have you ever been diagnosed with sarcoidosis, sarcoid‐like disease, or granuloma?” or for whom a review of their medical records indicated a diagnosis of sarcoidosis. In each case, a detailed medical record review was then carried out to determine level of certainty for the diagnosis of sarcoidosis.

“Definite” sarcoidosis was defined based on the following: a consistent clinical picture; noncaseating granulomas in at least one tissue sample; and exclusion of other causes of granulomatous disease.^3^ For example, in two cases with the potential for occupational exposure to beryllium (Be), negative results of Be lymphocyte proliferation tests (LPTs) were used to exclude chronic beryllium disease.

“Probable” sarcoidosis was defined as meeting the same criteria, with one exception: Although the medical record described biopsy‐confirmed sarcoidosis, there was no confirmatory histopathology report in the record. In most cases, the report was inaccessible because the biopsy was performed more than 25 years earlier. “Possible” sarcoidosis cases were those with diagnosis by a treating physician but a file that lacked sufficient confirmatory information. Cases were considered “unlikely” when alternative diagnoses had been made.

### 2.4 Exposure assessment

#### 2.4.1 Respirable crystalline silica

Occupational exposure information in the form of air sampling data came from three sources: the Mines Accident Prevention Association of Ontario semiannual reports of konimeter air sampling results from 1959 to 1975, gravimetric sampling data collected by the Ontario Ministry of Labour Training and Skills Development (MLTSD), and sampling data from individual mining companies. The Occupational Cancer Research Centre (OCRC) consolidated these data in the Ontario Mining Exposure Database (OMED), now containing over 118,000 sampling records.^27^ Approximately 65% of the records in OMED are for respirable dust, RCS, and radon. The remainder of the records are for a wide range of other contaminants in the mining environment. The OMED database has been developed further by OHCOW to make it searchable using a number of criteria (i.e., mine, type of contaminant, type of sample, task or location, etc.). Although some sampling data are available from the mid‐1950s, the majority of data are from 1970 to 1991. More than 4200 RCS measurements from gold, uranium, and nickel mines are contained in the OMED database. OMED was used as the source for our RCS exposure estimates.

Using detailed work histories and qualitative exposure information available for each miner from the intake questionnaires and confirmed by the Ontario MMF, we searched OMED by mine worked, job title and/or work tasks, and time period, and extracted RCS exposure data. A limitation of OMED is that sampling data are not available for all mines or for all periods worked by the cases. Where results for a specific mine and/or occupation or task were unavailable, overall data for the type of mine were used (e.g., all gold mines combined for the same job title or task and period). For the period 1950–1976, dust counts in ppcc were converted to mg/m^3^ using the conversion factors derived by Verma et al.^23^ Cumulative RCS exposures were calculated by multiplying the total number of years by the means and ranges of RCS exposure.

Latency was calculated by subtracting the year of initial RCS exposure from the year of sarcoidosis diagnosis.

#### 2.4.2 McIntyre Powder

Ontario gold and uranium miners were required to inhale MP for a prescribed time before each work shift. MP was administered at an airborne concentration of 35.6 mg/m^3^ in a custom‐designed airtight locker room (the mine dry) for 10 min before each shift, for an 8‐h time‐weighted average exposure of 0.74 mg/m^3^ (35.6 mg/m^3^ × 10 min/480 min).^24^, ^25^ The standardization of the MP aluminum prophylaxis program makes it likely that airborne concentration of MP was fairly constant across sites. MP was originally reported to be composed of 15% metallic aluminum and 85% aluminum oxide, but contemporary analysis of the powder has shown it to be primarily composed of aluminum hydroxide.^24^

#### 2.4.3 Other contaminants

Underground miners in Ontario were exposed to other airborne contaminants such as diesel exhaust, metal dust (i.e., zinc, copper), blasting agents, oil mist, and tungsten carbide. Although OMED contains some data on these contaminants, we determined that the data are too limited for use in quantitative estimates.^23^ However, these findings are noteworthy in a qualitative sense to underscore the range of exposures encountered by Ontario miners.

## 3 RESULTS

As of May 21, 2021, a total of 506 miners were registered and interviewed. Of these, 16 miners reported a history of sarcoidosis. In one additional case, initial review of the medical records obtained as part of the registration and intake process revealed a diagnosis of sarcoidosis by treating physicians. Health and work histories were obtained from the miners themselves in all but one case. In this case, because of the miner's diagnosis of dementia, information was obtained from his daughter who held Power of Attorney. Twelve cases were categorized as definite (*n* = 8) or probable (*n* = 4) sarcoidosis. Three were categorized as possible and 2 as unlikely. Data analysis was limited to definite or probable cases.

### 3.1 Demography and clinical findings

Demographic variables and smoking history are shown in Table 1. All are White males. Average age for the group as a whole is 74 years (range: 65–88 years). Distribution by smoking status at the time of sarcoidosis diagnosis is as follows: nonsmoker 2 (16.7%), former smoker 6 (50%), and current smoker 4 (33.3%).

**Table 1:** Demographics for miners with sarcoidosis

	Mean		Range
*Age (years)*			
Definite cases (*n* = 8)	75.6		72–88
Probable cases (*n* = 4)	70.8		65–78
Total	74		65–88
*Age at diagnosis (years)*			
Definite cases (*n* = 8)	57		42–76
Probable cases (*n* = 4)	33.8		29–45
Total	48.7		29–71

	Number	Percentage	
*Gender*		
Male	12	100%	
Female	0	0%	
*Race*			
White	12	100%	
Other	0	0%	
*Smoking status at diagnosis*			
Definite cases			
Nonsmoker	2		25%
Former smoker	6		75%
Current smoker	0		0%
Probable cases			
Nonsmoker	0		0%
Former smoker	0		0%
Current smoker	4		100%
Total cases			
Nonsmoker	2		16.7%
Former smoker	6		50%
Current smoker	4		33.3%

Clinical findings and workers' compensation status are shown in Table 2. Diagnosis of sarcoidosis was made based on mediastinal/hilar lymph node biopsy in 11 cases and lung biopsy in 1. The diagnosis was made in the usual course of medical care and not as a result of participation in Ontario's Ministry of Labour Mining Surveillance Program, which included periodic chest X‐rays, spirometry, and physical examination. In two cases, occupational history revealed the potential for Be exposure; BeLPTs were negative in both cases. Average age at diagnosis is 48.7 years (range: 29–71) for the group as a whole. Eleven had mediastinal/hilar lymph node involvement; and five, parenchymal disease. Five (41.6%) had extrapulmonary sarcoidosis, with clinical manifestations of disease in the endocrine system (hypercalciuria) in three (25%) and the heart in two (16.7%).^28^ None had evidence of mycobacterial or fungal infection. With the exception of polymyalgia rheumatica diagnosed in one case 26 years after sarcoidosis diagnosis, none had connective tissue disease. Family histories were negative for sarcoidosis.

**Table 2:** Clinical findings of miners with sarcoidosis

Case no.		1	2	3	4	5		6	7	8		9	10	11	12
Age at DX (years)		62	41	71	29	70		56	49	45		55	31	30	45
Smoking status at DX		ExS	ExS	ExS	CS	NS		NS	ExS	ExS		ExS	CS	CS	CS
Clinical presentation															
Symptoms		SOB	SOB	None	SOB/CP	None		Cough/SOB	Cough/CP	Cough/fever		None	SOB	None	None
Weight loss		No	No		No			No	Yes	Yes			No	No	No
Other		No	No		No			No	No	Arthralgias night sweats			No	No	No
Imaging results															
Hilar/mediastinal lymphadenopathy		Yes	Yes	Yes	UNK	Yes		Yes	Yes	Yes		Yes	Yes	Yes	Yes
Other adenopathy		No	No	No	UNK	Yes		No	No	No		No	No	No	No
Pulmonary fibrosis		Yes	Yes	No	Yes	Yes		Yes	No	No		No	No	Yes	No
Other parenchymal disease		Patchy opacities	No	No	UKN	No		No	No	No		No	No	No	No
Biopsy															
Tissue		LN	LN	LN	LUNG	LN		LN	LN	LN		LN	LN	LN	LN
Findings		NCG	NCG	NCG	NCG	NCG		NCG	NCG	NCG		NCG	NCG	No path	NCG
PFTS		Hyperinflation	OBSTR	OBSTR/LO DLCO	UKN	None		UKN	LO VC/DLCO	WNL		LO DLCO	UNK	UNK	WNL
X‐pulmonary disease		Hypercalcuria kidney stones	Nn	Cardiac	No	No		Hypercalcuria	No	No		No	Cardiac	No	Hypercalcuria
Alternative granulomatous disease															
Mycobacteria/fungal		NEG	NEG	NEG	NEG	NEG		NEG	NEG	NEG		NEG	NEG	UNK	UNK
BeLPT		NA	BeLPT NEG	NA	NA	NA		NA	BeLPT NEG	NA		NA	NA	NA	NA
Other															
FH		No	No	No	No	No		No	No	No		No	No	No	No
CtD		No	No	No	No	No		No	No	No		No	No	Nn	Nn
WSIB															
Confirmed/accepted		Yes/no	Yes/no	PEND	Yes/no	Yes/no		Yes/no	Yes/no	Yes/no		PEND	Yes/no	PEND	Yes/no

Abbreviations: BeLPT, beryllium lymphocyte proliferation test; CP, chest pain; CS, current smoker; DX, diagnosis; ExS, ex‐smoker; LN, lymph node(s); LO DLCO, low diffusing capacity of the lungs for carbon monoxide; LO VC, low vital capacity; NA, nonapplicable; NCG, noncaseating granuloma(s); NEG, negative; NS, never‐smoker; OBSTR, obstructive; PATH, pathology; PEND, pending; PFTS, pulmonary function tests; SOB, shortness of breath; UKN, unknown; WNL, within normal limits; WSIB, Workplace Safety and Insurance Board; X‐pulmonary disease, extrapulmonary disease.

Four of the 12 cases are described in detail below: three definite and one probable. These cases were selected to show examples of definite and probable cases, extrapulmonary sarcoidosis, and the single case of acute sarcoidosis. The numbering of cases is consistent in the text and in Tables 2, 3, 4. For Cases 1, 2, and 4, sarcoidosis diagnosis was confirmed by the WSIB, but workers' compensation was denied on the basis that sarcoidosis is not an occupational disease. In Case 3, the WSIB decision is pending.

#### 3.1.1 Case 1

This 73‐year‐old White male was diagnosed with sarcoidosis in 2010 at age 62. Two‐flight dyspnea was reported. Chest computed tomography (CT) scan showed mediastinal lymphadenopathy. Multiple subpleural nodules and patchy opacities 2–6 cm in size were described in the upper lung zones bilaterally. Pulmonary function tests (PFTs) showed mild hyperinflation. Bronchoscopy and cervical mediastinoscopy were performed, with biopsy of multiple lymph nodes. Pathology showed noncaseating granulomas with extensive fibrosis and obliteration of normal architecture. Extra‐pulmonary disease was present in the form of hypercalciuria. He was treated with prednisone.

Smoking status at the time of diagnosis is former smoker: 40–50 pack‐years with quit date in 1993.

Occupational history reveals work as an underground miner for more than 28 years. He worked in a gold mine for 6 years and a base‐metal mine for 22 years before diagnosis. Jobs in the gold mine included stope leader, slusher and electric scraper; and job activities, drilling, dynamite blasting, mucking, and rock bolting. His principal job in the base‐metal mine was skip tender, with responsibility for loading and unloading mine shaft cages with materials and personnel.

#### 3.1.2 Case 2

This 66‐year‐old White male was diagnosed with sarcoidosis in 1997 at age 41. Chest CT scan showed bilateral hilar lymphadenopathy. Mild shortness of breath was reported. Bronchoscopy and cervical mediastinoscopy were performed, with biopsy of a right paratracheal lymph node. Pathology showed noncaseating granulomas replacing much of the normal architecture. Shortness of breath worsened in 2009; PFTs showed moderate obstruction with preserved gas exchange. He was treated with prednisone. Chest CT scan in 2017 showed scarring of both upper lung zones and bilateral micronodules, as well as mild increase in size of right paratracheal and subcarinal lymph nodes.

Smoking status at the time of diagnosis is former smoker: 4 pack‐years with quit date in 1967.

Occupational history reveals that before sarcoidosis diagnosis the case worked in an asbestos mine for several months in 1973, and then at gold mines for 24 years: 7.25 years in a crusher plant on the surface and 15.75 years underground, primarily as a mechanic.

#### 3.1.3 Case 3

This 84‐year‐old White male was diagnosed with sarcoidosis in 2008 at age 71. CT scan showed right middle lobe atelectasis and a mass‐like area of increased density in the right hilum. He was asymptomatic; PFTs showed mild obstruction with mildly impaired gas exchange. Bronchoscopy and cervical mediastinoscopy with lymph node biopsies were performed. Pathologic interpretation was “Confluent sarcoid‐like granulomas.” Over the ensuing 5 years, he was monitored with periodic chest X‐ray/CT scans. In 2013, because of persistence of mediastinal lymphadenopathy and PET‐positive mass‐like lesion in the right hilum, bronchoscopy, mediastinoscopy, and lymph node biopsy were repeated, with similar findings: noncaseating granulomas replacing normal lymphoid tissue.

Cardiac sarcoid was diagnosed in 2008, 4 months after diagnosis of pulmonary sarcoid. Stent placement in the right circumflex artery in 2007 is the only evidence of pre‐existing atherosclerotic heart disease. In January 2018 he presented to a local hospital in atrial fibrillation without identifiable trigger. His condition deteriorated over the next several weeks and he developed congestive heart failure (CHF), with echocardiogram showing severe left ventricular (LV) global hypokinesis and ejection fraction (LVEF) < 25%–30% (normal ≥ 55%). With medical treatment that included prednisone, his condition improved and CHF resolved before successful cardioversion in mid‐July. In the context of his diagnosis of pulmonary sarcoidosis, his treating physicians attributed his LV dysfunction to cardiac sarcoid.

Smoking status at diagnosis is former smoker: 23 pack‐years of cigarettes with quit date in 1976, and occasional cigar and pipe‐tobacco use.

Occupational history reveals that the case worked underground in gold, uranium, and base‐metal mines for 28 years before his diagnosis of sarcoidosis: 2.8 years in gold mines, 10.5 years in uranium mines, and 8.5 years in nickel mines. Job activities included drilling, blasting, mucking, slushing, shaft sinking, and for a short period, construction work using air drills and tools.

#### 3.1.4 Case 4

This 65‐year‐old White male was diagnosed with sarcoidosis in 1985 at age 29. The presentation was acute with symptoms of chest pain and shortness of breath in June 1985 that worsened precipitously on July 5, resulting in his being taken by ambulance to a local hospital. Two weeks later he was transferred to a respiratory clinic for specialist evaluation and care. On August 1 he underwent surgical resection of the lower lobe of his left lung. Pathology showed multiple granulomas and a diagnosis of sarcoidosis was made. He was advised that the disease “would have to run its course” over the next 6–12 months. Symptoms resolved spontaneously and he was back at work in May 1987. Case 4 is designated as “probable” because of the lack of a confirmatory histopathology report in his record. The diagnosis of sarcoidosis was confirmed by the WSIB in December 1985 after review of the medical record by their consulting occupational medicine physician.

Smoking status at the time of diagnosis is current smoker: 1.5 purified protein derivative for 10 years with quit date in 1985.

At the time of symptom onset, the case had been working underground as a gold miner for 8 years. His principal job was stope miner, with activities that included drilling, slushing, timbering, shaft maintenance, construction, and “conventional cut and fill stopes with slushers and diesel scooptrams.” After his recovery, he worked for 20 years underground in gold mines and 9 years in surface operations at a base‐metal mine in training/supervisory and human resources positions, respectively.

### 3.2 Occupational exposures

#### 3.2.1 RCS

Table 3 summarizes the RCS exposure findings among our cases. The 12 sarcoidosis cases had an average exposure duration of 18.9 years (range: 6.0–28.5 years) for all types of mining combined. The average latency for the group as a whole is 29.2 years (range: 9–53 years).

**Table 3:** Summary of mining and RCS exposure^a^,^b^,^c^

**Cases**	**Gold**			**Base metals (e.g., nickel, copper, zinc)**			**Uranium**			**All mining**		
	**Total years** **(period)** **main job(s)**	**Annual** **mean RCS** **(range)** **mg/m** ^**3**^	**Mean** **cumulative RCS** **mg/m** ^**3**^ **years**	**Metal** **total years** **(period)** **main job(s)**	**Annual** **mean RCS** **(range)** **mg/m** ^**3**^	**Mean** **cumulative RCS** **mg/m** ^**3**^ **years**	**Metal** **total years** **(period)** **Main job(s)**	**Annual** **mean RCS** **(range)** **mg/m** ^**3**^	**Mean** **cumulative RCS** **mg/m** ^**3**^ **years**	**Total years RCS exposure**	**Annual** **mean RCS** **(range)** **mg/m** ^**3**^	**Total mean** **cumulative RCS** **mg/m** ^**3**^ **years**
Case no. 1	6.0 (1966–1972) stope miner (U)	0.04 (0.03–2.20)	0.24	Copper–zinc 22 (1972–1994) skip tender (U)	0.10 (0.03–0.16)	2.2	NIL	NIL	NIL	28.0	0.09 (0.03–2.20)	2.44
Case no. 2	4.0 (1974–1978) crusher operator (S) 18.0 (1979–1997) maintenance mechanic (S/U)	0.35 (0.03–2.08) 0.07 (0.01–0.88)	1.40 1.26	NIL	NIL	NIL	NIL	NIL	NIL	22.0	0.12 (0.01–2.08)	2.66
Case no. 3	2.8 (1983–1989) stope miner (U) driller (U)	0.03 (0.01–0.1)	0.08	Nickel 4.7 (1962–1972) stoper/driller (U) Nickel 3.8 (1992–1999) stoper/driller (U)	0.08 (0.01–2.41) 0.06 (0.03–0.17)	0.38 0.23	5.5 (1967–1977) driller (U) 5.0 (1978–1983) driller (U)	0.12 (0.02–3.24) 0.07 (0.01–0.73)	0.66 0.35	21.8	0.08 (0.01–3.24)	1.70
Case no. 4	8.0 (1976–1985) stope miner (U) drilling (U) scoop tram (U)	0.08 (0.01–0.88)	0.64	NIL	NIL	NIL	NIL	NIL	NIL	8.0	0.08 (0.01–0.88)	0.64
Case no. 5	4.3 (1964–1971) driller (U)	0.21 (0.01–3.40)	0.90	Copper/zinc 1.7 (1970–1973) driller (S)	0.05 (<0.01–>1.0)	0.09	NIL	NIL	NIL	6.0	0.17 (<0.01–3.40)	0.99
Case no. 6	28.5 (1966–1997) motorman (U) hoistman (U)	0.06 (0.01–0.28)	1.71	NIL	NIL	NIL	NIL	NIL	NIL	28.5	0.06 (0.01–0.28)	1.71
Case no. 7	2.8 (1967–1971) stope miner (U)	0.05 (<0.01–2.10)	0.14	Copper/zinc 10 (1971–1981) driller (U) stope miner (U) 15 (1981–1996) heavy equipment operator (S)	0.04 (<0.01–>5.0) 0.11 (0.01–1.80)	0.40 1.65	NIL	NIL	NIL	27.8	0.08 (<0.01–>5.0)	2.19
Case no. 8^c^	4.5 (1951–1955) surveyor (U)	0.44 (0.01–4.4)	1.00	Nickel 4.0 (1961–1965) surveyor (U)	0.10 (0.55–0.91)	0.20	5.5 (1955–1960) surveyor (U)	1.03 (<0.01–>8.92)	2.83	14.0	0.57 (0.03–2.20)	4.03
Case no. 9	26.0 (1967–1999) maintenance mechanic (U)	0.11 (<0.01–3.40)	2.86	NIL	NIL	NIL	NIL	NIL	NIL	26.0	0.11 (<0.01–3.40)	2.86
Case no. 10	4.7 (1962–1967) machine operator (U) 7.7 (1967–1974) mechanic (S)	0.06 (0.03–0.11) 0.18 (0.01–5.00)	0.28 1.40	NIL	NIL	NIL	NIL	NIL	NIL	12.4	0.13 (0.01–5.00)	1.61
Case no. 11	NIL	NIL	NIL	NIL	NIL		1.0 (1974–1976) drilling, blasting (U) 5.6 (1976–1983) dryman general maintenance (S)	0.08 (0.10–2.15) 0.09 (0.01–0.52)	0.08 0.50	6.6	0.09 (0.01–2.15)	0.58
Case no. 12	4.3 (1967–1972) mucking (U)	0.03 (0.01–0.04)	0.13	Zinc 1.0 (1972–1973) zinc refining (S) copper/zinc 20.0 (1973–1993) driller (U)	0.05 (0.10–0.18) 0.08 (0.01–0.11)	0.05 1.60	NIL	NIL	NIL	25.3	0.07 (0.01–0.18)	1.78

All cases	**Years mean** **(range)**	**Annual** **mean RCS** **(range)** **(mg/m** ^**3**^ **)**	**Mean** **cumulative RCS** **(range)** **mg/m** ^**3**^ **years**	**Years** **mean** **(range)**	**Annual** **mean RCS** **(range)** **(mg/m** ^**3**^ **)**	**Mean** **cumulative RCS** **(range)** **mg/m** ^**3**^ **years**	**Years** **mean** **(range)**	**Annual** **mean RCS** **(range)** **mg/m** ^**3**^	**Mean** **cumulative RCS** **(range)** **mg/m** ^**3**^ **years**	**Years** **mean** **(range)**	**Annual mean RCS** **(range)** **mg/m** ^**3**^	**Total mean cumulative RCS** **(range)** **mg/m** ^**3**^ **years**
	10.4 (2.8–28.5)	0.12 (<0.01–5.00)	1.10 (0.08–2.86)	13.7 (1.7–25.0)	0.08 (<0.01–5.00	1.13 (0.09–2.20)	7.5 (5.5–10.5)	0.41 (<0.01–8.92)	1.47 (0.58–2.83)	18.9 (6.0–28.5)	0.14 (0.06–1.13)	1.93 (0.64–4.03)

Abbreviations: RCS, respirable crystalline silica; S, surface; U, underground.

^a^ Work periods were typically not continuous (e.g., Case no. 1 worked at a gold mine for 4.25 years between 1964 and 1971).

^b^ Time weighted average concentrations for respirable crystalline silica for each type of mining were derived from Ontario Mining Exposure Database (see text).

^c^ Cumulative exposures for this case were adjusted to ½ the typical underground exposures as surveyors only spent about 50% of their time underground.

Gold mining was the most common type of mining, with all definite and three of four probable cases working an average of 10.4 years in gold mines. Five definite cases had an average of just over 12 years of exposure in base‐metal mines and two worked for 8 years in uranium mines. The annual mean RCS exposure for all cases combined was 0.14 mg/m^3^ (range: 0.06–1.13 mg/m^3^), and the mean cumulative exposure was 1.93 mg/m^3^ years (range: 0.64–4.03 mg/m^3^ years). The current Ontario MLTSD occupational exposure limit for RCS is 0.1 mg/m^3^ with a proposed lower limit of 0.025 mg/m^3^.

Among the cases, excessive RCS exposures were observed for surface as well as underground mining operations. For example, Case no. 7 had an annual mean RCS exposure of 0.35 mg/m^3^ during his 4 years of work in surface crushing operations that is comparable to Case no. 5 who had the highest annual mean RCS exposure of 0.44 mg/m^3^ during his 4.5 years of underground work in a gold mine.

In most cases, the range of RCS concentrations varied widely over the time periods worked at different mines. RCS concentrations in the mines generally declined over time as a result of regulatory changes and improvements in ventilation and other exposure control methods, such as the use of wet methods to reduce generation of dust during drilling and other work activities.

#### 3.2.2 McIntyre Powder

Table 4 summarizes MP exposures among our cases. Mean duration of exposure was 5.6 years (range: 2.3–12.2 years); and mean cumulative exposure, 4.2 mg/m^3^ years (range: 1.8–9.0 mg/m^3^ years).

**Table 4:** Summary of McIntyre Aluminum Powder exposure^a^

Case	Total years of exposure	Cumulative exposure mg/m^3^ years
1	5.6	4.1
2	5.4	4.0
3	3.5	2.6
4	2.3	1.8
5	3.2	2.4
6	10.3	7.6
7	3.0	2.2
8	5.9	4.4
9	4.9	3.6
10	12.2	9.0
11	5.8	4.3
12	5.5	4.1
All cases	Mean = 5.6, range: 2.3–12.2	Mean = 4.2, range: 1.8–9.0

^a^ Cumulative exposure calculated by multiplying total years of exposure by the calculated 8‐h TWA exposure of 0.74 mg/m^3^ as described by Blagove‐Hall et al.^27^

## 4 DISCUSSION

We present 12 cases of sarcoidosis in Northern Ontario hard‐rock miners. The cases were diagnosed over the period 1974–2017. We used conservative definitions of sarcoidosis, and present findings for definite and probable cases only.

Using provincial employment records, the OCRC matched 36,821 current and former hard‐rock miners with Ontario health database records covering the period between 1991 and 2018.^29^ Of these, close to 14,000 were exposed to MP during the course of their employment. Our sarcoidosis cases came from this group. To our knowledge, this is the first report of sarcoidosis in hard‐rock miners and the first to examine RCS exposures in detail.

### 4.1 Case characteristics

The cases in our series are unusual in certain respects. These include demography, smoking status, and clinical phenotype.

#### 4.1.1 Demography

Our cases are male and White; whereas sporadic cases of sarcoidosis in the general population occur at higher rates in women and in African Americans.^6^, ^30^ Gender and race likely reflect the Northern Ontario mining population from which they came, which is almost exclusively male and largely Caucasian.^29^ With regard to age at diagnosis, our cases resemble the overall population averages reported by Arkema and Cozier.^31^

#### 4.1.2 Smoking status

Smoking appears to have a protective effect with regard to risk for sarcoidosis in the general population.^31^, ^32^ Most of our cases were current or former smokers at the time of diagnosis. Jonsson et al.^13^ in their study of a cohort of silica‐exposed Swedish construction workers observed an increase in risk among ever‐smokers and no increase in risk among never‐smokers. A potentially confounding factor is the selection of sarcoidosis cases from the national in‐patient register, as smokers are more likely than nonsmokers to be hospitalized.

#### 4.1.3 Extrapulmonary sarcoidosis

An estimated 30%–50% of sarcoidosis cases in the general population have extrapulmonary involvement.^6^, ^33^ Close to 42% of our cases had extrapulmonary sarcoidosis determined on the basis of medical record review that revealed histologic evidence of noncaseating granulomas, without other cause, in at least one organ system and clinical manifestations of disease, without other cause, in at least one other organ system. Although these criteria are consistent with the sarcoidosis organ assessment tool developed by the World Association of Sarcoidosis and Other Granulomatous Diseases, a standardized instrument was not used in assessing extrapulmonary sarcoidosis in our series of cases^34^

An estimated 2%–5% of sarcoidosis patients in the general population have clinical manifestations of cardiac sarcoid.^35^, ^36^ In our case series, 2 of 12 (16.7%) cases were diagnosed with cardiac sarcoid. Both had CHF, with global LV hypokinesia and reduced LVEF in the absence of clinical evidence of ischemic heart disease (IHD). In one of the two cases, IHD was specifically ruled out by normal coronary angiogram. Both had arrhythmias, in one case atrial and in the second case, atrial and ventricular. These observed findings are consistent with clinical manifestations of cardiac sarcoid and were without other evident cause.^35^, ^36^

Hena et al.^16^ observed nine (16%) cases of cardiac sarcoid in a group of 59 New York City firefighters exposed to WTC dust in 2001 and followed for 15 years with detailed medical monitoring that included screening with cardiac magnetic resonance imaging. None of these cases was observed at the time of diagnosis of pulmonary sarcoidosis. The proportion of cases with cardiac sarcoid in our case series is similar to that observed in WTC‐exposed firefighters and higher than that observed in the general population. Unlike our cases, NYC firefighters were screened with cardiac MRI's. Hena et al.^16^ do not distinguish between those cases identified by clinical manifestations and those identified by screening, if any.

Twenty‐five percent of our cases had evidence of endocrine sarcoid, compared to 10%–30% of the general population.^28^ None of the NYC firefighters had abnormal calcium metabolism.

#### 4.1.4 Type of presentation

Acute presentation of sarcoidosis was observed in one of our cases. Consistent with the more typical presentation in the context of Lofgren's syndrome (LS), symptoms resolved spontaneously within a 2‐year period of time.^37^ Human leukocyte antigen (HLA)‐type genes have been associated with increased risk for LS.^37^, ^38^ Certain non‐HLA genes have been associated with a different phenotype that more closely matches that of our case: acute self‐limiting disease without the skin and joint manifestations of LS.^38^ HLA testing was not done in our case.

### 4.2 Occupational exposures

We have examined RCS exposures for the cases by duration and mean and cumulative exposure. Vihlborg et al.^14^ and Graff et al.^15^ used similar parameters to assess exposure in their respective cohort and case‐control studies of sarcoidosis in populations of Swedish workers exposed to RCS. Vihlborg et al.^14^ determined air concentrations of RCS using personal sampling data collected in 10 iron foundries; and Graff et al.,^15^ using an updated job‐exposure‐matrix created using the Swedish Occupational Register.

Vihlborg et al.^14^ and Graff et al.^15^ observed a positive exposure–response relationship between RCS and risk for sarcoidosis.^17^, ^18^ In the cohort study of iron foundry workers, a significant increase in standardized incidence ratio (SIR) was observed at annual mean RCS exposures ≥ 0.048 mg/m^3^: SIR: 3.94, 95% confidence interval (CI): 1.07–10.08.^14^ As shown in Table 3, all cases in our case series had annual mean RCS exposure concentrations > 0.048 mg/m^3^, with a range of 0.06–1.13 mg/m^3^.

In their case‐control study of sarcoidosis incidence in a more diverse group of Swedish workers, Graff et al.^15^ observed a significant increase in disease risk with increasing duration of silica exposure in the age group >35 years: for duration 6–<11 years, odds ratio [OR]: 1.28, 95% CI: 1.03–1.59; for duration ≥11 years, OR: 1.44, 95% CI: 1.04–2.00.^15^ Stratification by age at diagnosis revealed increased overall risk in the exposed age group ≤35 years compared to the exposed age group >35 years: OR: 1.48, 95% CI: 1.1–1.87 versus OR: 1.21, 95% CI: 1.05–1.39, respectively. No consistent increase in sarcoidosis risk was observed in association with mean or cumulative silica exposures.

A commonly held assumption is that surface mining operations have much lower RCS exposures than those in underground mining. However, the OMED data and our findings show that surface operations such as milling or refining generate RCS concentrations that are similar to or greater than those of underground operations.

The effect of MP exposure on risk for sarcoidosis is unclear. As an aluminum powder, MP may have contributed directly to granuloma formation in the cases in our series. Peters et al.^39^ conducted a mortality study of Australian gold miners exposed to MP in the 1950s and 1960s for silicosis prophylaxis. Although sarcoidosis was not examined as an outcome, no excess death from pneumoconiosis was observed.

Aluminum exposure in refinery and production workers has been associated with pulmonary fibrosis and granuloma formation.^40^ The incidence of granulomatous reactions in the lungs of aluminum‐exposed workers was described by Hoyle^41^ as “very low.” In 1978 Chen et al.^42^ reported a case of pulmonary granulomatosis associated with occupational exposure to aluminum dust. Electron probe microanalysis of lung tissue identified the presence of aluminum. In 1987 De Vuyst et al.^43^ reported a case of sarcoid‐like granulomas in the lungs of a 32‐year‐old chemist who worked in a catalyst fabrication plant with exposure to aluminum metal and oxide powders. Clinical work‐up for sarcoidosis, including Kveim test, was negative. Aluminum LPT using peripheral blood lymphocytes was positive. Analytical electron microscopy of lung tissue obtained by transbronchial biopsy revealed aluminum particles within the granuloma cells. In a report of two cases of granulomatous lung disease in a battery‐manufacturing worker and an aluminum‐processing worker, Tomioka et al.^44^ attributed the lung disease to aluminum exposure based on elemental analysis showing aluminum widely distributed in the granulomas.

We did not have the opportunity to conduct elemental analyses of granulomatous tissue in our cases. We hope to investigate the specific contribution of aluminum‐containing MP in a case‐control study of sarcoidosis in the population of hard‐rock miners in Ontario, comparing prevalence of sarcoidosis in MP‐exposed miners to prevalence in miners not exposed to MP.

### 4.3 Gene‐exposure interactions

Grunewald et al.^38^ have proposed a model for the development of sarcoidosis and for sarcoidosis phenotypes based upon genetics and gene‐exposure interactions. The model requires the existence of “susceptibility genes,” with the development of disease only in the context of certain exposures. Others have described the importance of gene‐exposure interactions on risk for the development of sarcoidosis.^45^, ^46^ For example, in a case‐control study of sarcoidosis in NYC firefighters exposed to WTC dust, Cleven et al.^47^ found 17 allele variants of HLA and non‐HLA genes in cases that were not present in WTC‐exposed firefighter controls without sarcoidosis. Less attention has been paid to clinical phenotypes. Cardiac sarcoid has been associated with HLA‐DQB1*0601; multiorgan involvement with non‐HLA gene CCL5/RANTES,17q.12; and acute self‐limiting sarcoidosis (vs. chronic sarcoidosis) with the non‐HLA gene TGF‐β2,1q41.^38^ Gene‐exposure interactive effects on such clinical manifestations have not been reported to our knowledge. Effects of such interactions on risk for disease and on clinical phenotype are hypothetical at the moment and require additional research in exposed populations for further clarification and verification.

## 5 CONCLUSIONS

Our observation of 12 sarcoidosis cases in a group of 506 Northern Ontario hard‐rock miners is consistent with and provides support for the associations between occupational exposure to RCS and sarcoidosis reported in the scientific literature. Our case series is unique in its detailed examination of occupational histories specific to each case, with quantification of cumulative RCS exposure based on air sampling data from mines in which they worked. Neither our case series nor published reports demonstrate a causal relationship between RCS exposure and sarcoidosis, but both inform our assessment of the association between the two.

Questions raised by this case series are several: (1) What are the interactive effects of RCS, smoking, and genotype on risk for sarcoidosis; (2) how may these interactive effects influence clinical phenotype; and (3) what is the contribution of MP to the development of sarcoidosis in these miners?

That it is time to discard the habit of referring to sarcoidosis as idiopathic should not be in question. Some cases of sarcoidosis do not have an identifiable cause. Others do. The key to disease prevention, proper treatment, and just workers' compensation is recognizing the difference.

## CONFLICTS OF INTEREST

L. Christine Oliver is a medical consultant to OHCOW; her work related to this case series was carried out as part of her overall consulting work with the organization. Janice Martell founded the McIntyre Powder Project and is the daughter of a miner who was exposed to McIntyre Powder. She had no direct involvement in, or influence over the interpretation of medical findings or exposure information. The remaining authors have no conflicts of interest to declare.

## DISCLOSURE BY AJIM EDITOR OF RECORD

John Meyer declares that he has no conflict of interest in the review and publication decision regarding this article.

## AUTHOR CONTRIBUTIONS

L. Christine Oliver and Andrew M. Zarnke conceptualized the idea of publication of a case series based upon data in the miner cohort. L. Christine Oliver reviewed and interpreted all medical information, developed the criteria for case definition, and took the lead in writing the manuscript. Paul Sampara reviewed, organized, analyzed, and interpreted industrial hygiene sampling data and took the lead in writing the occupational exposure sections of the manuscript. Donna Pearson performed nursing summaries of medical findings and occupational histories for the cases, contributed to specific aspects of mining job and exposure descriptions, and developed tables presenting the results. Janice Martell initiated subject recruitment and data collection as part of the McIntyre Powder Project, contributed to the methods, and conducted outreach to cases to obtain informed consent. Andrew M. Zarnke contributed to the methods and interpretation of findings, as well as overall organization of the writing of the manuscript.

## ETHICS APPROVAL AND INFORMED CONSENT

There was no requirement for ethics or institutional review and approval because this study was not experimental. Informed consent for publication was obtained from all living cases. For each of the three deceased cases, informed consent was obtained from the executor of their estate.

## DISCLAIMER

The opinions and conclusions expressed are solely those of the authors.

## Data Availability

The data that support the findings of this study are available on request from the corresponding author. The data are not publicly available due to privacy or ethical restrictions.
